# Genotyping by Sequencing Reveals Genetic Relatedness of Southwestern U.S. Blue Maize Landraces

**DOI:** 10.3390/ijms22073436

**Published:** 2021-03-26

**Authors:** Amol N. Nankar, Richard C. Pratt

**Affiliations:** 1 Center of Plant Systems Biology and Biotechnology (CPSBB), 4000 Plovdiv, Bulgaria; 2 Department of Plant and Environmental Sciences, New Mexico State University, Las Cruces, NM 88003-8003, USA; ricpratt@nmsu.edu

**Keywords:** biodiversity, crop diversity, germplasm conservation, germplasm utilization, blue maize, genotyping-by-sequencing, SNP-Type

## Abstract

Maize has played a key role in the sustenance and cultural traditions of the inhabitants of the southwestern USA for many centuries. Blue maize is an important component of the diverse landraces still cultivated in the region but the degree to which they are related is unknown. This research was designed to ascertain the genotypic, morphological, and phenotypic diversity of six representative southwestern blue maize landraces. Their genotypic diversity was examined using tunable genotyping-by-sequencing (tGBS™). A total of 81,038 high quality SNPs were identified and obtained through tGBS. A total of 45 morphological and biochemical traits were evaluated at two locations in New Mexico. The varieties Los Lunas High and Flor del Rio were genetically less related with other southwestern landraces whereas diffusion between Navajo Blue, Hopi Blue, Yoeme Blue, and Taos Blue demonstrated that these landraces were genetically related. Phenotypic variability was highest for kernel traits and least for plant traits. Plant, ear, and kernel traits were fairly consistent within and across locations. Principal component analysis and tGBS showed that Corn Belt variety ‘Ohio Blue’ was distinctly different from southwestern landraces. Genotypic analysis displayed that southwestern landraces are genetically closely related, but selection has resulted in differing phenotypes. This study has provided additional insight into the genetic relatedness of southwestern blue maize landraces.

## 1. Introduction

The development of modern cultivars and farming systems narrows the germplasm base and heightens crop genetic vulnerability [1,2]. Less genetic diversity will restrict our capacity to maintain and enhance crop production and our ability to respond to climate change. Plant genetic resources are vital assets for improving human conditions and crop diversity must be preserved to ensure global food security. Landraces are the primary contributors to the diversity of our genetic resources. They are essential in traditional farming systems, conventional or modern breeding, and genetic engineering programs. Seed banks play a vital role in the preservation of genetic diversity, and so too does conserving landraces in situ.

Historically, the richness of in situ crop genetic diversity has been protected within “cultural landscapes”. In Mexico, the birthplace of maize [3], Hernandez [4] envisioned the landscape as a three-way relationship between environment, culture, and maize. Sadly, roughly 80% of the genetic diversity of maize has already been lost. Fortunately, substantial reservoirs of crop diversity remain in certain regions where landraces are still used in traditional farming systems. These unique areas can be termed hotspots [5] or “primary regions of diversity” [6]. Identification, characterization, and preservation of the remaining crop genetic resources in hotspots is urgently needed.

The southwestern region of the USA (the Southwest) is such a relatively unknown hotspot for traditional cultivation of diverse maize landraces. Archeological evidence confirms that native communities of the Southwest have practiced maize farming for more than 4100 years [5,7,8]; making it the oldest continuously managed agricultural area in the USA. Hunter-gatherers during the Basketmaker Era gradually adopted races introduced from the Mexican Highlands. These races became adapted to semi-arid agricultural systems [9] across large elevational gradients, and new races evolved over millennia with periodic influxes of different varieties and races from Mexico [7,10,11,12] and through trade with Southern Plains Indian tribes [13]. An era of rapid development of maize cultivation in the Southwest occurred starting approximately 2000 years ago, a time that also coincided with an influx of Chapalote and Reventador races that came to the Southwest through Pacific Coastal routes in present Sinaloa, Mexico [7]. Pre-colonial southwestern landraces assumed paramount importance in the native farm communities that cultivated, conserved, and maintained them for generations [14].

Increasingly dynamic movement, exchange, and interaction of diverse crop germplasm likely occurred with the arrival of new farming cultures in the Southwest during the Spanish Colonial and U.S. Territorial eras. The increasing movement of peoples and seed made possible by improvements in transportation likely facilitated a higher frequency of genetic exchange between races resulting in the occurrence of racial admixtures. Anderson and Cutler [15] noted the presence of what they referred to as “recent admixtures” and some obvious “intermediates” between Pima-Papago and Pueblo races. A north-south pathway of gene influx along the eastern piedmont of the Sierra Madre Occidental has also been proposed by Hernández and Flores [13] who described the similarities between the newly identified northern race of Maiz Azul (Blue Maize) in northern Mexico and Puebloan maize from New Mexico, and also between the races Blandito de Sonora and floury Papago (Pima-Papago) maize [13,16].

Sturtevant [17] noted that 18 of 18 samples of maize cultivated by Native American Indians in Arizona and New Mexico displayed soft (floury) kernels. That author also commented on the diverse colors of southwestern maize varieties, notably samples from the Zuni and Tesuque Pueblos of New Mexico. Anderson and Cutler [15] stated that Pueblo maize is usually colored and Pima-Papago maize is either white or a bright light yellow. Blue maize can still be found throughout the Pueblos of New Mexico, and it is also of special importance to the Hopi of northern Arizona [18]. Blue maize is also grown on Navajo farms in Arizona, New Mexico, and Utah, but as Nabhan [19] pointed out, Navajo blue floury maize can look remarkably different from Hopi blue floury maize grown just a few miles away.

Today, blue maize is highly valued by southwestern Hispanic and Native American communities, especially in northern New Mexico. It also appears with lower frequency in other parts of the Southwest, e.g., in the homelands of the Yoeme (southern Arizona and northern Sonora, Mexico). It is not represented among the predominant landrace (Pima-Papago) of the Pima and Tohono O-odham tribes of Arizona. Of the two major southwestern races, Pima-Papago is considered to be relatively uniform phenotypically, whereas Pueblo maize may display traits that have resulted from recent influxes of races such as Southeastern and Southern Dent or even Corn Belt Dent [20,21]. Doebley et al. [22] examined the taxonomic and anthropological implications of diverse landraces of southwestern maize and concluded that Pima-Papago and Puebloan maize differed in isozyme constitution, but showed some overlap, suggesting gene exchange between races that associated with geographically isolated cultural systems. Papago maize displayed little isozyme variation within landraces, but much among landraces. Pueblo maize showed considerable variation within landraces, but less among the landraces.

Maize landraces are open-pollinated varieties from which farmers save seed for subsequent planting. They are not static populations since they continue to evolve in response to farmer-directed and natural selection for adaptation to local physical, social, and cultural environments within a particular geographical region [23,24,25]. Traditional farmers have retained these landraces for their particular storage, cooking, nutritional, and processing qualities, as well as for historical and cultural reasons. Those reasons may include a desire for traditional foods, dietary diversity, fulfilling market niches or use in religious ceremonies [26,27].

Use of specific landrace varieties is typically associated with perceptual distinctiveness (PD) traits, which serve as indicators for identification and maintenance of landrace integrity [28]. These traits can assist in maintaining the genetic purity between diverse landraces suited for planting at particular locations or for various end-uses. In northwestern Mexico, the PD trait of kernel color has been popularly used by farmers as an ecological, dietary, and medicinal indicator [26]. The southwestern blue maize varieties likely reflect the same or very similar PD trait selection. It is assumed that Southwestern blue maize does not constitute a race such as Maiz Azul (Blue Maize) in the isolated high-altitude regions of Chihuahua, Mexico. Rather, traditional farmers in different geographic regions may have independently recognized blue kernel color as a PD trait reflecting their preference for its intrinsic value or as an indicator of ecological or cultural value. In this case, nomenclatural aggregation of southwestern landraces of blue maize could be useful primarily for similar end-use product differentiation.

The PD traits, which allow recognition of individual landraces by farmers, can also be used by taxonomists to create and manage racial diversity [28,29]. The need for natural classification, and difficulties associated with grouping maize into natural races and sub-races, was discussed by Anderson and Cutler [15]. Classical studies contributed fundamental principles for racial classification based on morphological traits [30,31] and natural classification [32,33,34]. Because morphological traits are influenced by environmental factors, and because many interacting genes often contribute to trait expression, morphological diversity is not an ideal measure of genetic diversity. Variability for ear morphology traits can make classification of maize accessions across regions difficult [35] but their relationship to PD traits used by farmers makes them relevant. We wished to determine whether natural classification groups could be achieved using the different trait data, or if genetic diversity would be displayed by southwestern blue maize varieties expressing the same PD trait (anthocyanin based blue/purple kernel color). We examined representative landraces of southwestern blue maize using molecular, morphological, and biochemical descriptors from replicated test sites in New Mexico.

## 2. Results

Tunable genotyping by sequencing (tGBS) of blue southwestern maize landraces was performed to identify their genetic relatedness. We also examined morphological and biochemical traits previously used to classify maize races and sub-races to allow comparison with tGBS findings. The genotypic analysis (tGBS) showed degree of relatedness among the southwestern landraces, but all (except Navajo Blue) were unrelated to the Corn Belt variety ‘Ohio Blue’. Morphological and kernel compositional traits ascertained phenotypic and biochemical variation. The highest variability between southwestern landraces was observed for kernel traits and the lowest for plant traits.

### 2.1. Genotypic Diversity

#### 2.1.1. SNP Discovery and Identifying Genetic Polymorphisms

Several sets of single nucleotide polymorphisms (SNPs) were generated during tGBS analysis. The first set of 1,437,967 polymorphic sites included all sites that differed from the reference in at least one sample. This set was generated after all reads that aligned to the reference genome. Then we examined, sample-by-sample and identified a set of 217,178 “ALL SNPS”, which aligned to the polymorphic sites. Subsequently, ALL SNPs were further filtered and a subset of high-quality SNPs was identified that had less than 50% missing data (low missing data or LMD50) across the 105 individuals. The resulting LMD50 (each of which was genotyped in at least 50% of the samples) SNP set contained 81,038 SNPs.

**Figure 1:**
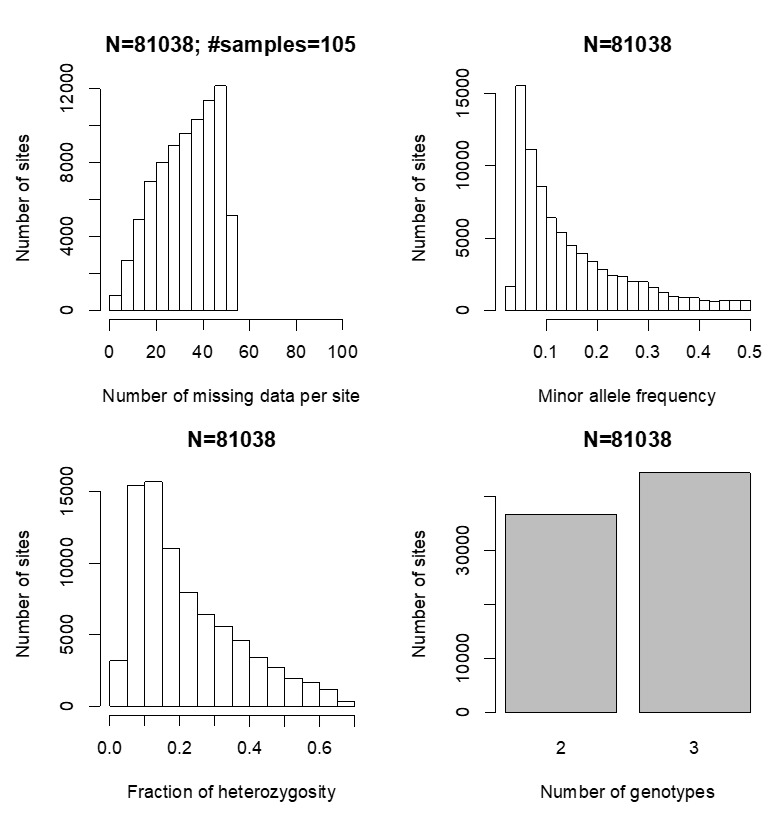
Site-based, low missing data (LMD) single nucleotide polymorphisms SNPs genotype summary. Distribution of missing data, minor allele frequency, heterozygosity, and genotype number are used to describe the LMD SNPs summary.

**Figure 2:**
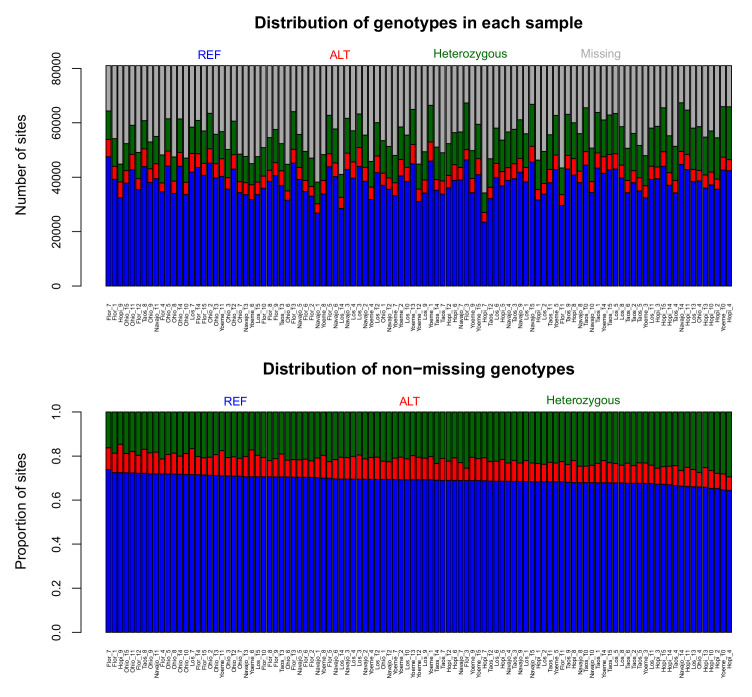
Sample-based, low missing data (LMD50) SNPs genotype summary. LMD50 refers to low missing data, each of which was genotyped in at least 50% of the samples. The top panel shows the summary of SNP genotypes includes the number of SNPs those are homozygous for the REF allele (reference allele), homozygous for the ALT allele (alternate allele), and heterozygous among missing data. The bottom panel shows the proportions of the SNPs per sample that are homozygous for the REF allele, homozygous for the ALT allele, or heterozygous among the non-missing data.

**Figure 3:**
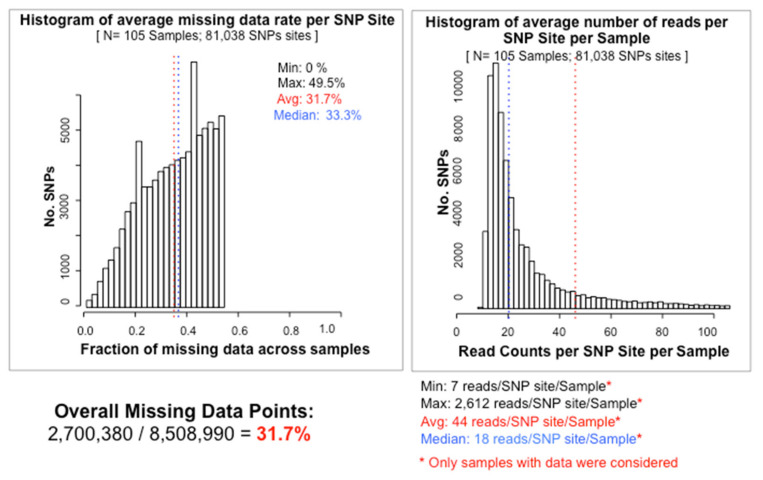
LMD50 SNPs average missing rate per SNP and read counts per SNP site per sample. The average missing data rate per LMD50 SNP site across samples is provided in the left panel and minimum, maximum, average and median number of reads per SNP per sample are shown in the right panel.

Distributions of various characteristics for the LMD50 SNPs dataset, including quantity of missing data, minor allele frequency, heterozygosity and genotype number, are summarized in Figure 1. In the tGBS analysis, a total of 81,038 high quality SNPs were identified, each of which exhibited less than 50% missing data among the 105 samples used to create the phylogenetic tree. The summary of SNP genotypes including the number of SNPs that are homozygous for the REF allele (reference allele), homozygous for the ALT allele (alternate allele), and heterozygous and missing data, can be seen in the top panel of Figure 2. The bottom portion of the panel shows the proportions of the SNPs per sample that are homozygous for the REF allele, homozygous for the ALT allele, or heterozygous among the non-missing data. The average missing data rate per LMD50 SNP site across samples is provided in the left panel in Figure 3. Sequencing data support 68.3% of all possible SNP calls. The right panel of Figure 3 presents the minimum, maximum, average and median number of reads per SNP per sample. Each SNP call was supported by 44tGBS sequence reads per sample, thus ensuring the accuracy of these non-imputed SNP calls.

#### 2.1.2. Population Structure Analysis

**Figure 4:**
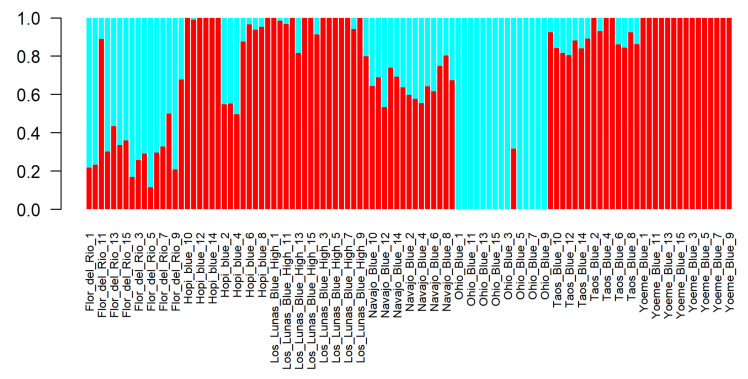
Barplot displaying genetic structure distribution of two groups (K = 2) identified based on lowest cross-validation (CV) error. Group 1 comprised of blue maize landraces of southwest origin and group 2 consists of Midwestern Ohio Blue.

Based on 81,038 SNPs from 105 individuals of seven accessions, the population structure within southwestern blue maize landraces was investigated. We ran the Admixture software with K ranging from 1 to 10 in the assumptions of 1 to 10 sub-populations defined in the studied genotypes. By the cross-validation error (CV error) among K = 1 to 10, the minimum CV error was detected at K = 2 (Figure S1) and consequently, the population was divided in two groups including six and one genotypes, respectively (Figure 4). Group 1 comprised of Navajo Blue, Los Lunas High Blue, Flor del Rio, Yoeme Blue, Hopi Blue, and Taos Blue landraces, which are of Southwest origin. Group 2 consists of Midwestern Ohio Blue (Figure 4). The population structure of K = 1 to 10 is shown in Figure S2, which shows the presence of sub-population in each population structure analysis such as one sub-population in K = 1, two sub-populations in K = 2 and so forth until K = 10. The percent contribution of each sub-population in respective population structure analysis is shown in Table S1. The structure analysis has validated the distinct separation of Ohio Blue from southwestern blue maize landraces as seen in the phylogenetic analysis and principal component analyses.

#### 2.1.3. Phylogenetic Analysis

**Figure 5:**
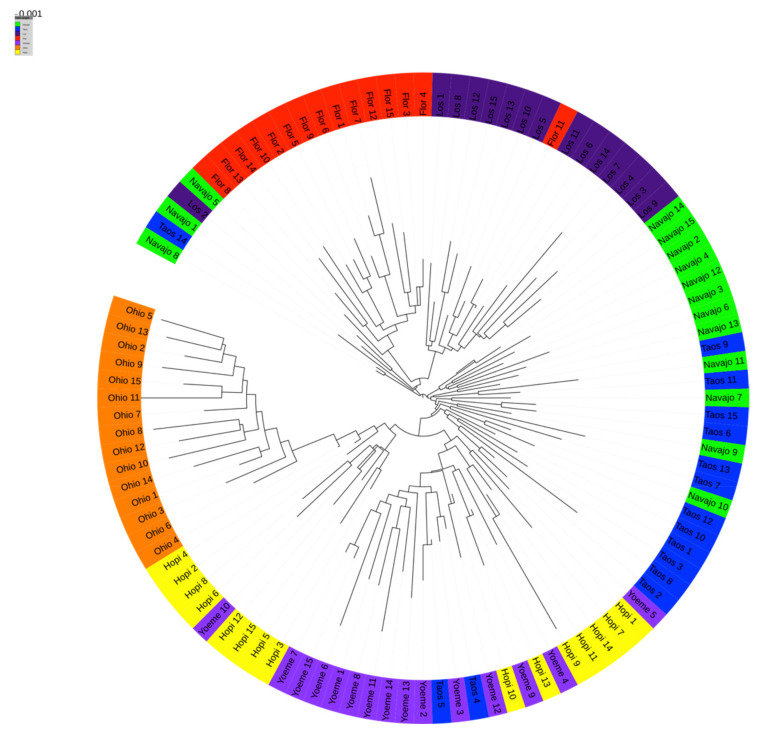
Phylogenetic tree of sequenced genotypes. Phylogenetic tree was built using 81,038 high quality SNPs, which exhibited less than 50% missing data among the 105 samples.

The phylogenetic tree of all 105 sequenced genotypes is shown in Figure 5 which displays the evolutionary relationships between the studied landraces. The phylogenic tree is categorized with landraces using different colors. All samples from a given accession (one color) are grouped together or at most two landraces are mingled within the tree. The phylogenetic tree further validates the distinct difference of monophyletic group Ohio Blue from all southwestern landraces as it represents evidence of independent development of Ohio Blue without genetic exchange with other landraces in the phylogeny. Flor del Rio and Los Lunas High were also grouped separately from the rest of the landraces. There was evidence of genetic exchange between individual plants of Flor del Rio and Los Lunas High populations. The paraphyletic group of Navajo Blue, Hopi Blue, Taos Blue, and Yoeme Blue were mixed, amongst all four accessions and discerns evidence of genetic exchange between these landraces. Hopi Blue was more variably distributed than the rest of the landraces. The genetic exchange of this accession showed that Hopi Blue and Yoeme Blue were genetically related with each other. Taos Blue and Navajo Blue are also closely related to each other.

### 2.2. Phenotypic Trait Variation

#### 2.2.1. Quantitative Trait Variation

In phenotypic evaluations, pre-harvest plant and post-harvest trait diversity were discerned through detailed examination of variation related to agro-morphological features of plant, ear, and kernel descriptors (Table 1). Trait variation was examined at different phenological growth stages ranging from vegetative, reproductive to post-harvest stage. Landraces evaluated across locations displayed significant differences for post-harvest ear and kernel traits while location effect showed significant differences for pre-harvest plant traits only (Table 1). However, the interaction between accession and location (A*L) showed non-significant differences for all traits except for kernel rows per ear and grain yield (Table 1). Across locations, the highest range of variation was observed for number of tillers (35.4%), ear height (22.2%), number of secondary branches (20.9%), number of ears per plant (20.1%), and grain yield (19.8%) (Table 1) while least variation was for circumference of ear middle (4.0%), circumference of cob bottom (3.4%), and cob diameter (4.0%). The detailed morphological characteristics of plant, ear and kernel traits for Los Lunas are shown in Tables S2, S4, and S6, respectively. Most traits evaluated at Los Lunas showed broad variability for plant (Table S2), ear (Table S4), and kernel traits (Table S6). The morphological traits associated with plant, ear, and kernel traits evaluated at Alcalde are shown in Tables S3, S5, and S7, respectively. Interestingly, most plant (Table S3), ear (Table S5), and kernel (Table S7) trait values obtained from Alcalde were relatively higher as compared to those from Los Lunas.

#### 2.2.2. Qualitative Trait Variation

The relative proportion of color classes for different morphological traits and presence of kernel dent phenotype between different blue maize landraces is shown in Table 2. A Chi-square analysis of these qualitative traits is shown in Table 3. The majority of tassels, silks and glumes, across all landraces were green, except those of Flor del Rio. Some purple, white, and a combination of purple and green colors were also observed (Table 2). Most landraces displayed white and green leaf midribs and shoots, respectively. The Flor del Rio accession was distinctly more variable than other landraces with both green and purple tassels and red, purple, brown and/or white cobs. Chi-square analysis of these qualitative traits showed that tassel, silk, glume, leaf midrib, shoot and cob color classes were significantly different among landraces (Table 3) evaluated at each location as well as across both locations.

#### 2.2.3. Kernel Compositional Trait Variation

The descriptive statistics for kernel biochemical traits across locations are shown in Table 4. Detailed descriptive statistics for Los Lunas and Alcalde are shown in Table S8 and S9, respectively. In all evaluated landraces, Taos Blue displayed the highest oil and fatty acid contents whereas Flor del Rio showed the lowest. Highest protein content was reported from Flor del Rio and lowest from Los Lunas High and Taos Blue (Table 4). Starch content was invariably similar across all landraces except Yoeme Blue. Anthocyanin content was highly varied, and Santa Ana Blue and Hopi Blue displayed highest anthocyanin values whereas Flor del Rio displayed the lowest. Santa Ana Blue and Taos Blue consistently showed higher values for most of the biochemical traits, whereas Flor del Rio displayed the lowest values, except for protein content. Location-wise, kernel biochemical traits evaluated at Los Lunas (Table S8) were higher than those from Alcalde (Table S9), except starch.

### 2.3. Principal Component Analysis (PCA)

**Figure 6:**
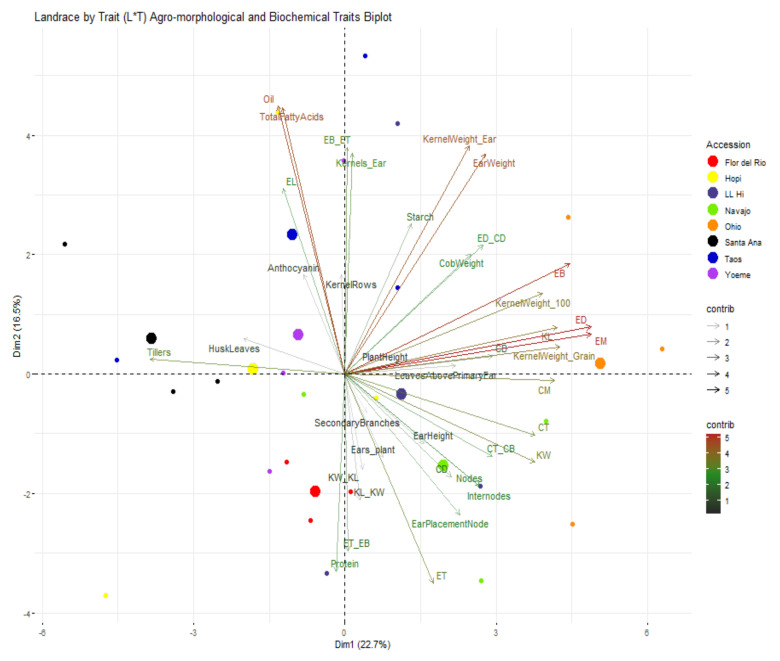
Landrace by trait (L*T) biplot for agro-morphological and biochemical traits. Phenotypic diversity for different morphological traits was calculated based on total of 34 agro-morphological traits including pre-harvest plant traits and post-harvest ear and kernel traits and 5 biochemical traits. Some traits shown in this figure have been abbreviated and full forms for the abbreviated traits are presented in Table 5. Landraces from Navajo Blue, LL High, Santa Ana Blue, Flor del Rio, Yoeme Blue, Ohio Blue, Hopi Blue, and Taos Blue are shown in “chartreuse2”, “slateblue4”, “black”, “red1”, “darkorchid2”, “darkorange”, “yellow” and “blue3”, respectively. Traits contributing to PC1 and PC2 are also assigned different colors with a gradient ranging from 1,3, and 5 with “gray15”, “forestgreen”, and “firebrick”, respectively. Larger consensus points for each accession discerns the midpoint of a given accession.

In order to assess the morphological and biochemical diversity between southwestern blue maize landraces we examined the traits in groupings that could reflect associated PD traits. In this manner, pre- and post-harvest morphological traits were also examined at the specific plant organ level. PCA analyses were then performed on pre-harvest plant traits, post-harvest ear and kernel traits, and kernel biochemical traits. The variation contributed by these traits can be seen in Figure 6. The variability generated for different agro-morphological and biochemical traits were contributed by a total of 23 principal components (PCs). The first 11 PCs with > 1 eigenvalue were identified by factor analysis and around 92.39% of the total phenotypic variance was contributed by these PC (Table 5) and first two components explained 22.69% (PC1) and 16.50% (PC2) variance, respectively. Landrace by trait (L*T) biplot between PC1 and PC2 displayed number of tillers, circumference of ear mid and bottom, ear diameter, circumference of cob top and mid, kernel length, kernel width, and kernel weight as the primary contributing traits to PC1 variance (Figure 6 and Table 6); whereas kernel compositional traits (total fatty acids, protein, and oil), number of kernels per ear, kernel weight per ear, ear length, ear weight, circumference of ear top, ratio between circumference of ear top and bottom, and ratio between circumference of ear bottom and ear top contributed to the PC2 variance. Biplot between PC1 and PC2 showed that the traits associated with PC1 distinctly separated Ohio Blue and Flor del Rio and those associated with PC2, separated Santa Ana Blue, and Yoeme Blue from the rest of the southwestern landraces (Figure 6). Hopi Blue, LL High, and Yoeme Blue were observed to be the most phenotypically variable landraces.

**Figure 7:**
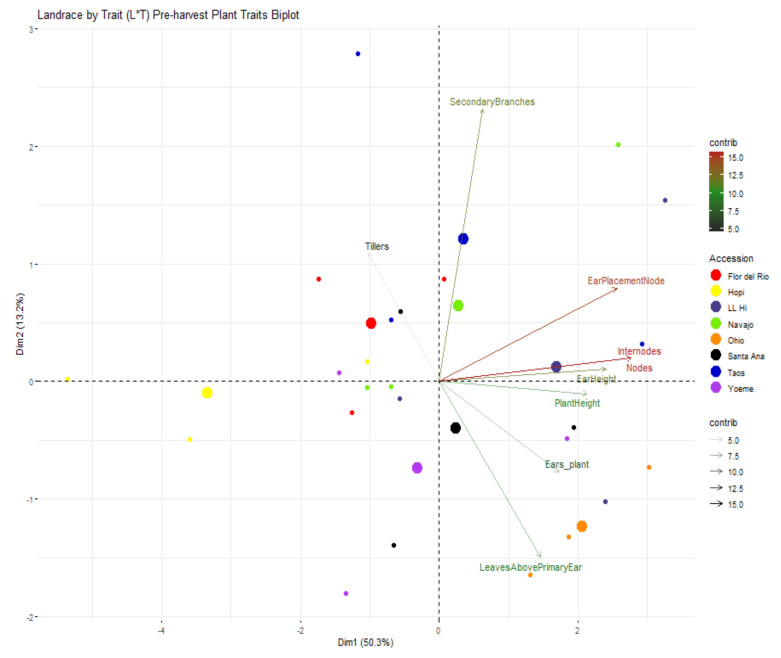
Landrace by trait (L*T) biplot for pre-harvest plant traits. Landraces from Navajo Blue, LL High, Santa Ana Blue, Flor del Rio, Yoeme Blue, Ohio Blue, Hopi Blue, and Taos Blue are shown in “chartreuse2”, “slateblue4”, “black”, “red1”, “darkorchid2”, “darkorange”, “yellow” and “blue3”, respectively. Traits contributing to PC1 and PC2 are also assigned different colors with a gradient ranging from 5, 10, and 15 with “gray15”, ”forestgreen” and “firebrick”, respectively. Larger consensus points for each accession discerns the midpoint of a given accession.

**Figure 8:**
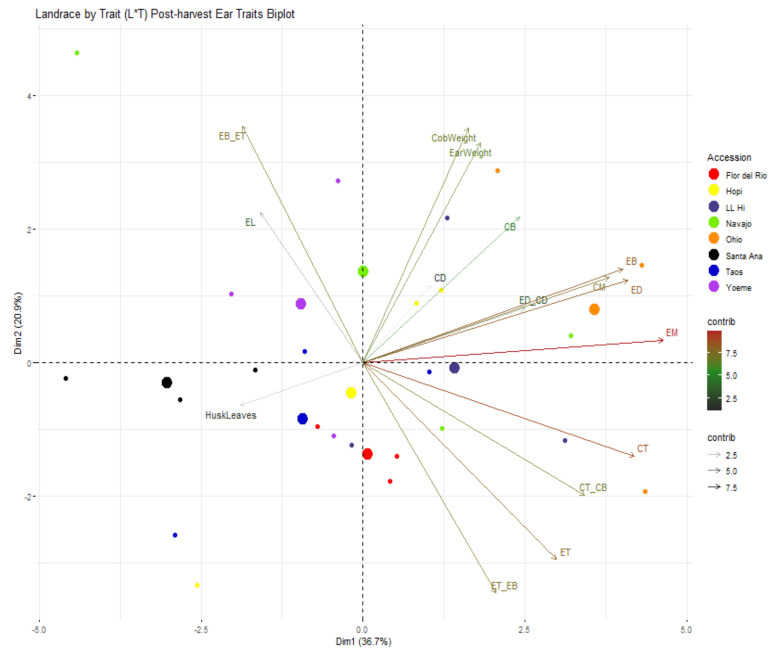
Landrace by trait (L*T) biplot for post-harvest ear traits. Some traits shown in this figure has been abbreviated and full forms for the abbreviated traits are presented in Table 5. Landraces from Navajo Blue, LL High, Santa Ana Blue, Flor del Rio, Yoeme Blue, Ohio Blue, Hopi Blue, and Taos Blue are shown in “chartreuse2”, “slateblue4”, “black”, “red1”, “darkorchid2”, “darkorange”, “yellow” and “blue3”, respectively. Traits contributing to PC1 and PC2 are also assigned different colors with a gradient ranging from 2.5, 5, and 7.5 with “gray15”, ”forestgreen” and “firebrick” respectively. Larger consensus points for each accession discerns the midpoint of a given accession.

Variability contributed by different plant traits can be seen in Figure 7 where 63.5% of variability was contributed by PC1 and PC2. The variability contributed to PC1 was predominantly due to plant height, ear height, ears per plant, leaves above primary ear, number of nodes and internodes whereas variability contributed to PC2 was due to number of tillers, and secondary branches (Figure 7). Based on variability of plant traits, Ohio Blue appeared distinctly different from the rest of the southwestern landraces (Figure 7). The variability for ear traits can be seen in Figure 8 where 56.7% of variability was contributed by PC1 and PC2. Circumference of ear (mid and bottom), circumference of cob (top and bottom), and ear diameter mainly contributed the variability to PC1 whereas ratios between circumference of ear top and bottom and circumference of ear bottom and top and cob weight contributed to the variability of PC2 (Figure 8). The majority of southwestern landraces were similar in terms of variation of ear traits except Santa Ana Blue; however, Ohio Blue differed from the southwestern landrace cohort except for partial overlap with Navajo Blue (Figure 8).

**Figure 9:**
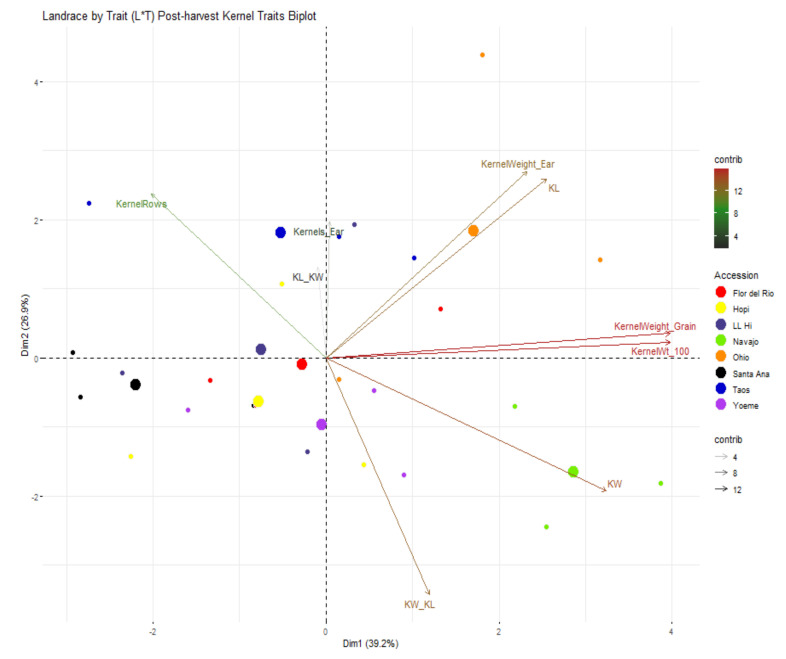
Landrace by trait (L*T) biplot for post-harvest kernel traits. Some traits shown in this figure has been abbreviated and full forms for the abbreviated traits are presented in Table 5. Landraces from Navajo Blue, LL High, Santa Ana Blue, Flor del Rio, Yoeme Blue, Ohio Blue, Hopi Blue, and Taos Blue are shown in “chartreuse2”, “slateblue4”, “black”, “red1”, “darkorchid2”, “darkorange”, “yellow” and “blue3”, respectively. Traits contributing to PC1 and PC2 are also assigned different colors with a gradient ranging from 4, 8, and 12 with “gray15”, ”forestgreen” and “firebrick” respectively. Larger consensus points for each accession discerns the midpoint of a given accession.

**Figure 10:**
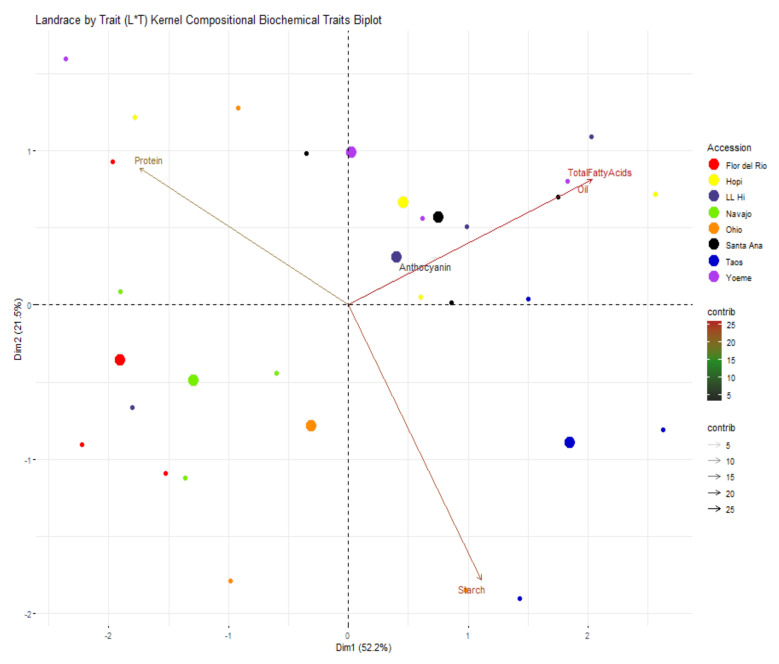
Landrace by trait (L*T) biplot for kernel biochemical traits. Landraces from Navajo Blue, LL High, Santa Ana Blue, Flor del Rio, Yoeme Blue, Ohio Blue, Hopi Blue, and Taos Blue are shown in “chartreuse2”, “slateblue4”, “black”, “red1”, “darkorchid2”, “darkorange”, “yellow” and “blue3”, respectively. Traits contributing to PC1 and PC2 are also assigned different colors with a gradient ranging from 5, 15, and 25 with “gray15”, ”forestgreen” and “firebrick” respectively. Larger consensus points for each accession discerns the midpoint of a given accession.

Diversity associated with kernel traits was mainly related to post-harvest kernel morphological and biochemical traits. PCA biplot for kernel traits is shown in Figure 9 where 66.1% of variance was contributed by PC1 and PC2. Ratio between kernel width and length, kernel width, 100 kernel weight, and single kernel weight contributed to PC1 whereas kernel rows per ear, number of kernels per ear and ratio between kernel length and width contributed to PC2 variability. Group-wise, Navajo Blue separated from other landraces; however, Ohio Blue was overlapped with Flor del Rio and Taos Blue (Figure 9). Biochemical diversity was analyzed using kernel biochemical traits and a total of 73.5% of variability for biochemical traits was contributed by PC1 and PC2 (Figure 10). Variability for PC1 was mainly contributed by starch whereas PC2 variability was contributed by protein. Biochemical diversity estimated using PCA displayed no distinct grouping as all landraces overlapped.

## 3. Discussion

Genotypic, morphological, and biochemical traits were used to determine the genetic diversity and relatedness of southwestern U.S. blue maize landraces. The landraces were representative of different geographic regions of the Southwest, and a Corn Belt Dent variety was included for comparison. The relatedness of Hopi Blue, Yoeme Blue, Taos Blue, Los Lunas High and Navajo Blue suggests that there has been a common origin, with some gene flow between distinct regional landraces. Noteworthy is the east-west diffusion between Taos Blue and Navajo Blue and between Yoeme Blue and Hopi Blue across northern and southern Arizona/northern Sonora, Mexico. Overall findings suggest that the southwestern landraces are genetically closely related, but selection has resulted in differing phenotypes. A key finding of our study was the dissimilarity of natural classifications achieved by phenotypic and genotypic analysis. Conclusions regarding groupings will vary depending on the type of analysis, number of traits evaluated in a given analysis, and uncontrolled variation accrued from sample size, individual trait attributes (i.e., their heritability), and environmental factors.

Genotypic analysis was based on 81,043 high quality SNPs whereas phenotypic analysis was conducted using 40 diverse morphological traits. The disproportionate number of traits could have been a major factor for these differences. Evaluation of morphological traits included plant, ear and kernel traits. Kernel traits were more variable than the plant and ear traits, therefore our findings showed similarity for some traits whereas dissimilarity among others. The genotypic analysis was done based on the genetic sequences and none of the variation was accrued from environmental factors, which were likely a major source of variation in phenotypic analysis. Our findings, taken together provide a fuller picture of both the genotypic and phenotypic relatedness between the landraces.

The findings from our PCA analysis for morphological traits showed a variation of 57.7%, 14.1%, and 11.7% due to PC1, PC2, and PC3, respectively. These values were lower than those observed by Sánchez et al. [36] which suggests that racial classification across multiple environments and years is more robust than those based on a single year evaluation. The biochemical diversity variation of PC1, PC2, and PC3 was reported at 59.0%, 39.8%, and 1.1%, respectively. Racial classification using biochemical traits has not been reported in the recent past. The Principal Coordinate Analysis (PCA) of Doebley et al. [25] study showed no distinct clusters among different southwestern landraces. Significant overlap was reported among them. Our study also showed overlap between different landraces―with the exception of Navajo Blue. Our results are also consistent with the presence of interracial admixtures among Pueblo maize varieties from New Mexico [11].

Hernandez and Flores [13] studied similar morphological traits from Mexican Maiz Azul (Mexican Blue) race with the exception of shank length, which we did not examine. The plant height reported in our study ranged from 1.6 to 2.0 m in comparison to 1.9 to 2.2 m plant height for Maiz Azul. The number of nodes and internodes reported in our study ranged from 13 to 14, respectively in comparison to reported eight to nine nodes in Maiz Azul. Seventeen secondary tassel branches were reported in our study whereas 2 to 3 secondary tassel branches were observed in Maiz Azul. Tassels of Maiz Azul appear to be considerably smaller than those of southwestern blue maize landraces. Ear traits measured in southwestern blue maize were closely aligned with Hernandez and Flores [13] findings of Maiz Azul morphological traits. Average ear and cob diameter reported from southwestern blue maize landraces were 3.9 and 2.7 cm, respectively and Hernandez and Flores [13] also reported similar observations for Maiz Azul. Blue kernel color reported in southwestern blue maize was similar to Maiz Azul kernel pigmentation. The differences observed showed that the variation in plant, ear and kernel characteristics might be mainly associated with geographical and sociocultural differences involved in the traditional cultivation and farmer selection. The Maiz Azul race is found in Western Mexico and is cultivated by Mestizos tribes in the mountainous region of western Chihuahua whereas blue maize landraces found in the Southwest are grown by different American Indian tribes from New Mexico and Arizona.

The qualitative traits of tiller, silk, glume, midrib, shoot and cob color of blue maize have not been studied previously for racial classification with the exception of the Soleri and Smith [18] study. Those authors studied the glume color and have reported red and purple glumes from Hopi Blue whereas we have reported green and purple/green glumes, which suggest that we were examining different landraces, both called Hopi Blue. Beside aesthetic importance of qualitative traits, kernel color in pigmented maize has played a pivotal role in selection for nutrition and socio-cultural importance in the Southwest USA for centuries [26,27]. The importance of kernel colors in selection for human nutrition has presumably “de novo” evolved in the Southwest [37] and may have created different races based on the kernel color.

Sánchez and Goodman [38] classified Mexican races using cluster analysis and identified three different racial groups. A sub-group from the Sierra de Chihuahua group containing several races of maize from the highlands of central and northern Mexico was also revealed. The sub-groups included the Cristalino de Chihuahua, Gordo, Azul (Blue), Apachito, and Serrano de Jalisco. Those races are restricted to the highlands of northwestern Mexico in valleys from 2000 to 2600 m above sea level. Maiz Azul is characterized by short-statured plants with few tassel branches and long slender ears that are tapered at the base. Kernels are rounded and tend to be of floury texture. In contrasts, samples of blue maize from the Southwest display considerable variation for plant and ear characteristics, and they are cultivated in diverse environments at a broad range of altitudes [20]. The racial classification of southwestern maize by Adams et al. [24] was based on 27 distinct groups of 123 pigmented maize landraces and a total of four groups were formed using PCA based on the ear length and shank size. Our results were based on a broader scale including the evaluation of pre-harvest phenotypic characters, post-harvest morphological traits, and kernel biochemical traits. Based on the PCA, Corn Belt Dent Ohio Blue was readily distinguished from the southwestern landraces.

Diversity of germplasm collections can be studied at phenotypic or morphological, geographical, molecular, and functional levels [39]. Genotypic diversity analysis has allowed us to understand the genetic similarities and differences between southwestern landraces and a distant Corn Belt population, Ohio Blue.

## 4. Materials and Methods

### 4.1. Germplasm

**Figure 11:**
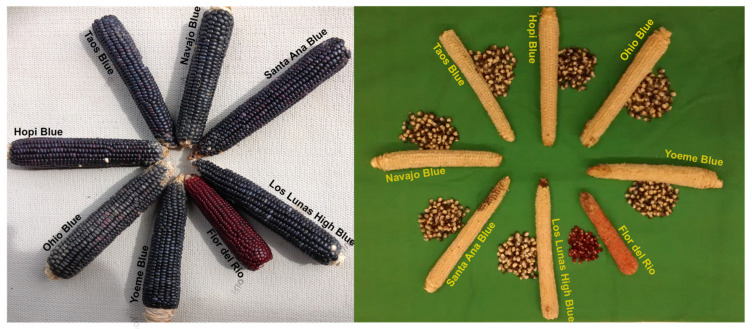
Representative ears (unshelled-on **left**) and kernels with cobs (shelled-on **right**) of blue corn landraces. All landraces are labeled and are of Southwestern US origin except Midwestern Corn Belt Ohio Blue.

We examined six blue maize landraces representative of the blue maize found in Arizona and New Mexico. The six landraces and two improved open-pollinated populations were evaluated during the 2014 field study. The southwestern landraces: Taos Blue (NS/S ZM03-015), Yoeme Blue (NS/S ZM01-011), Hopi Blue (NS/S ZM02-147), and Flor del Rio (NS/S ZP-093 Popcorn), were made available through Native Seeds/SEARCH (NSS) (Tucson, AZ). Navajo Blue was obtained from Plants of the Southwest (Santa Fe, NM), Santa Ana Blue, and Los Lunas “High” populations were contributed by the New Mexico State University (NMSU) Agricultural Science Center, Los Lunas, NM. Los Lunas “High” was selected from Santa Ana Blue and possibly other Puebloan blue maize varieties. For comparison Ohio Blue, a Corn Belt Dent variety derived from Blue Clarage and Ned’s Blue, was obtained from the Ohio Agricultural Research and Development Center, Ohio State University, Wooster, OH. Clarage is the oldest documented landrace in Ohio and was once widely cultivated in central and northern Ohio [40]. Other related types of clarage include ‘Improved Clarage’, ‘Eichelberger Clarage’, and ‘Rotten Clarage’ [40,41]. Ned’s Blue was still offered for sale by Ned Place of Wapakoneta, OH in Auglaize County (western Ohio) until the early 21st century. It was apparently selected from local corn and was grown in the Ohio, Indiana, and Michigan region. Its origin is unknown, but it is conceivable that it was also selected from Rotten Clarage (a mixture of blue, yellow, and mixed pigmentation kernels) that was popular in southwestern Ohio. Geographical and botanical features of each accession can be seen in Table 7 and cultivation region of each landrace is shown in Figure S3. Representative ears and cobs (shelled ears) with kernels of each accession are shown in Figure 11.

### 4.2. Experimental Location

During 2014, blue maize landraces were planted at NMSU Agriculture Research Centers at Los Lunas and Alcalde, New Mexico. A randomized complete block design with three replications was used. Seeds were planted in 6.1 m long plots with planting distance of 0.45 and 0.61 m between hills (2 seeds per hill) at Los Lunas and Alcalde, respectively. Los Lunas is located in Valencia County, NM and is situated at latitude of 32.28 and longitude of −106.76 with elevation of 1480 m. Alcalde is located in Rio Arriba County, NM and is situated at 36.68 latitude and −106.05 longitude with elevation of 1741 m.

### 4.3. Trait Measurement

#### 4.3.1. Genotypic Diversity Analysis

##### Genotyping-By-Sequencing

A total of 105 samples representing 15 plants from each of six open-pollinated varieties of southwestern blue maize, and one of Corn Belt blue maize, were genotyped with Data2Bio’s tunable genotyping by sequencing technology [42].

##### Trimming of Sequencing Reads

Prior to alignment, the nucleotides of each raw read were scanned for low quality bases. Bases with PHRED quality value <15 (out of 40) [43,44], i.e., error rates of ≤3%, were removed by our trimming pipeline. Each read was examined in two phases. In the first phase reads were scanned starting at each end and nucleotides with quality values lower than the threshold were removed. The remaining nucleotides were then scanned using overlapping windows of 10 bp and sequences beyond the last window with average quality value less than the specified threshold were truncated. The trimming parameters were referred to the trimming software, Lucy [45,46].

##### Alignment of Reads to Public Maize B73 Reference Genome

Trimmed reads were aligned to the Maize reference genome using GSNAP [47] and confidently mapped reads were filtered if it mapped uniquely (≤ 2 mismatches every 36 bp and less than 5 bases for every 75 bp as tails) and used for subsequent analyses.

##### Discovery of Polymorphic Sites

The coordinates of confident and single (unique) alignments to the consensus reference sequence that passed our filtering criteria were used for SNP discovery. Polymorphisms at each potential SNP site were carefully examined and putative homozygous and heterozygous SNPs were identified in each sample separately using the following criteria:

- Homozygous SNP calling
  ○ The most common allele was supported by at least 80% of all the aligned reads covering that position.
  ○ At least 5 unique reads supported the most common allele.
  ○ Polymorphisms in the first and last 3 bp of each read were ignored.
  ○ Each polymorphic base had at least a PHRED base quality value of 20 (≤ 1% error rate).
- Heterozygous SNP calling
  ○ Each of the two most common alleles was supported by at least 30% of all aligned reads covering that position.
  ○ At least 5 unique reads supported each of the two most common alleles.
  ○ The sum of reads of the two most common alleles accounted for at least 80% of all aligned reads covering that nucleotide position.
  ○ Polymorphisms in the first and last 3 bp of each quality-trimmed read were ignored.

Each polymorphic base had at least a PHRED base quality value of 20 (≤1% error rate).

##### Population Structure Analysis

Population genetic structure was analyzed using Admixture software version 1.3.0 [48]. Admixture has become mainstream software for genetic population structure analysis by virtue of its high-speed computation. Prerequisite conditions of Hardy-Weinberg equilibrium and minor allele frequency (MAF = 0.05) were met in the analyzed data set. The independent SNPs were selected by the –show-tags all and –block in plink software [49,50] before subjecting to structure analysis. The SNPs without any high-correlated SNPs (r2 = 0.8) in the data set and the first SNPs in each haplotype block were kept as the independent SNP data set. The binary files of SNPs and the assumed number of sub-population (K = 1 to 10) applied to the Admixture. Cross-validation error (CV error) was extracted from the results file. CV error rate of different K values was used to identify the best K value based on the smallest CV error. The obtained results were further visualized in R to obtain the final distribution of CV error using package ggplot2 version 3.3.3 [51] as shown in Figure S1. The results showed that K = 2 corresponds to the smallest CV error hence identified as the best K value. According to the results file calculated by the software Admixture at K = 2, the genetic structure bar plot was created using biplot R package.

##### Phylogenetic Tree Construction

Pairwise distances were estimated between genotyped individuals using an unbiased model of substitution frequencies. Distance estimates were then used to construct a phylogenetic tree using the Neighbor-Joining like algorithm described by Criscuolo and Gascuel [52] and implemented in the njs module of the R APE package [53]. Unlike conventional neighbor joining methods, the njs algorithm is tolerant of missing data, enabling its use with GBS data. Relative branch lengths are proportional to the amount of divergence observed among individuals.

#### 4.3.2. Measurement of Phenotypic Traits

A total of 40 morphological traits were examined (Table S10). Pre-harvest traits were measured when the plants were standing in the field at two New Mexico locations (Los Lunas and Alcalde) in 2014. Ear and kernel traits were measured post-harvest in the laboratory. Ear length was measured using a measuring board and ear diameter, cob diameter, kernel length, and kernel diameter was measured using calipers (General^®^ ULTRATECH, Secaucus, NJ, USA). Weights of ear, cob, kernel, and 100-kernel samples were measured using a NewClassic MS Balance (Mettler Toledo, Columbus, OH, USA).

#### 4.3.3. Measurement of Biochemical Traits

Five kernel biochemical traits were analyzed from representative kernel samples produced at Los Lunas and Alcalde in 2014. The Experiment Station Chemical Laboratory of the University of Missouri analyzed the kernel constituents. The traits of total fatty acids, oil, protein, starch, and anthocyanin were examined. Oil and protein were measured by AOAC method 920.39 (A) and 990.03. Starch content was analyzed using base method: American Association of Cereal Chemists, approved method 76-13 and total fatty acids were analyzed by AOAC official methods 996.06 and Ca 5b-71. Total anthocyanin content was analyzed according to Li et al. [54] and Nankar et al. [55] method.

### 4.4. Statistical Analysis

Analyses of variance for both pre- and post-harvest traits were performed for each location separately as well as interaction between accession and location (A*L) was also evaluated using pooled data across both experiment locations. Analysis of variance of quantitative traits was performed using “PROC GLM” and Chi-Square analysis of qualitative traits was performed using “PROC FREQ”. Landraces were considered as fixed effects and locations were considered as random effects. Means were compared by least significant differences test using “LSD” mean separation. Statistical analysis was performed with SAS V9.3 [56]. Principal component analysis of phenotypic diversity was performed on a total of 34 morphological and five kernel biochemical traits using R program version 4.0.3. Eigenvalue, eigenvector, percent variance of different principal components, and accession by trait biplot were estimated by *ggplot2* version 3.3.3 [51], *missMDA* version 1.18 [57], *FactoMineR* version 2.4 [58], and *Factoextra* version 1.0.7 [59] R packages.

## 5. Conclusions

In this research, we have employed data on morphological, biochemical, and molecular variation to characterize the genetic diversity of southwestern blue maize landraces. The use of a molecular technique tGBS was more effective than morphological or biochemical traits for determining distinct varieties among southwestern blue corn landraces. The coalesced analysis of genetic structure, phylogeny, and principal component analysis proved to be effective in elucidating genetic structure of southwestern blue maize landraces from the midwestern Ohio Blue variety. However, the majority of southwestern landraces appeared to display interracial diffusion and belong to the same cohort, with the exceptions of Los Lunas High and Flor del Rio. Among the southwestern blue maize landraces, Navajo Blue displayed noticeable variation whereas Santa Ana, Los Lunas High, Flor del Rio, Yoeme Blue, Hopi Blue and Taos Blue showed little variation. Weight of cob, ear, kernel, 100 kernels and kernels per ear contributed to the variability in Navajo Blue. The groupings were more robust when performed using post-harvest traits. Our findings confirm that southwestern blue maize landraces are genetically related, and reflect the attributes of admixtures, but are phenotypically uniform. Diversity of trait values suggested that selection for a strong PD (blue kernel color) did not result in uniformity for other traits, but that overlap in phenotypic traits was consistent with earlier evidence of genetic exchange between southwestern landraces of maize.

## Figures and Tables

**Table 1:** Descriptive statistics and analysis of variance (ANOVA) for all pigmented maize landraces evaluated by pre-harvest plant and post-harvest ear, and kernel traits evaluated across Alcalde and Los Lunas. Level of significance expressed is * *p* < 0.05, ** *p* < 0.01; *** *p* < 0.001.

Trait (Unit)	Code		Across Locations							
		Descriptive Statistics				ANOVA				
							F-Value			
		Mean	Range	LSD_0.05_	CV		Accession	Replication	Location	A*L Interaction
						DF	7	2	1	7
**Pre-Harvest:**										
**Plant Traits**										
Plant height (cm)		180.9	157.7–200.7	27.7	12.8		1.8	1.6	71.3 ***	1.5
Ear height (cm)		81.9	68.6–90.3	21.4	22.2		1.0	0.4	19.3 ***	0.8
Ears/plant		2.0	1.9–2.1	0.5	20.1		0.2	1.1	5.6 **	1.1
Number of tillers		3.1	2.3–3.8	1.3	35.4		1.8	0.1	11.3 ***	2.0
Secondary branches		17.1	14.9–20.4	4.2	20.9		1.3	1.3	15.8 ***	1.4
Leaves above primary ear		6.4	6.2–6.8	0.7	8.8		0.7	0.1	22.9 ***	0.9
Number of nodes		13.9	13.5–14.8	1.4	8.2		0.8	1.1	41.4 ***	1.5
Number of internodes		12.9	12.5–13.8	1.4	8.9		0.8	1.1	41.4 ***	1.5
Ear placement node		6.6	6.3–7.0	1.0	13.3		0.5	2.4	25.9 ***	1.2
**Post-Harvest:**										
**Ear Traits**										
Circumference of ear top (cm)	ET	9.7	8.0–10.7	1.2	9.0		3.9 *	4.4 *	15.1 ***	1.5
Circumference of ear middle (cm)	EM	12.9	12.0–14.0	0.6	3.9		7.5 ***	0.3	10.4 **	0.4
Circumference of ear bottom (cm)	EB	13.6	13.0–14.8	0.7	4.2		5.4 ***	0.4	0.6	0.7
Circumference of cob top (cm)	CT	6.1	5.5–6.6	0.7	9.1		2.2	2.9	4.9 **	0.5
Circumference of cob middle (cm)	CM	8.3	7.8–8.6	0.4	4.1		3.7 **	0.1	2.9	0.3
Circumference of cob bottom (cm)	CB	9.3	8.9–9.6	0.4	3.4		3.0 **	0.5	14.5 ***	1.4
ET/EB		0.7	0.6–0.7	0.1	8.8		1.5	1.8	13.5 ***	1.5
CT/CB		0.7	0.6–0.7	0.1	8.4		1.8	2.5	15.4 ***	0.9
Ear length (cm)	EL	21.9	20.4–24.2	2.2	8.4		2.4 **	0.6	0.4	1.3
Ear diameter (cm)	ED	4.0	3.80–4.5	0.9	18.0		0.7	1.0	0.1	1.2
Cob diameter (cm)	CD	2.7	2.56–2.8	0.1	4.0		2.7 **	5.7 **	2.7	0.42
ED/CD		1.5	1.42–1.7	0.3	17.4		0.9	2.1	0.1	1.2
Ear weight (gm)	EW	160.7	135.75–188.9	26.5	14.0		3.1 **	0.3	4.6 **	1.1
Cob weight (gm)	CW	30.3	26.10–33.9	5.2	14.7		2.7 **	0.3	10.7 **	1.1
Number of husks		10.4	8.72–11.5	1.8	14.3		2.2	2.5	12.3 ***	0.5
Kernel rows per ear		14.2	13.17–14.7	1.7	7.0		1.6	0.1	1.3	2.8 **
Number of kernels per row		39.3	36.28–42.9	4.0	8.6		2.1	0.2	0.7	2.1
Number of kernels per ear		492.6	437.1–550.8	61.2	10.5		2.9 **	2.3	3.1	2.1
**Kernel Traits:**										
Kernel length (cm)	KL	1.1	1.1–1.2	0.1	5.3		3.9 **	0.6	0.2	0.5
Kernel width (cm)	KW	0.8	0.7–0.9	0.1	6.2		5.2 ***	1.9	3.7	0.9
KL/KW		1.3	1.2–1.4	0.2	11.4		1.0	9.4 ***	3.9 **	0.2
KW/KL		0.7	0.7–0.8	0.1	4.9		4.3 **	3.2 **	2.7	1.8
Kernel weight (gm)		0.3	0.3–0.3	0.1	8.3		6.0 ***	0.4	2.1	1.4
100-Kernel weight (gm)		26.7	23.6–29.9	2.6	8.2		6.6 ***	0.4	1.8	1.4
Grain yield (mg/ha)		3.0	2.8–3.6	0.7	19.8		1.3	0.4	17.4 ***	2.7 **

**Table 2:** Relative proportion of different color classes for morphological traits and presence of kernel color phenotype among different blue corn landraces.

Trait	Color Class	Navajo Blue	Santa Ana Blue	Los Lunas High	Flor del Rio	Yoeme Blue	Ohio Blue	Hopi Blue	Taos Blue
**Tassel Color**	**Green**	83.3	58.3	58.3	50	75	75	100	100
	**Purple**	16.7	33.3	41.7	33.2	16.7	8.3	-	-
	**Purple (Green)**	-	-	-	8.4	-		-	-
	**Other**	-	8.4	-	8.4	8.3	16.7	-	-
**Silk Color**	**Green**	90.9	95.8	77.3	66.7	91.6	95.6	80.9	100
	**Purple**	4.5	-	4.5	-	-	-	4.8	-
	**Purple (Green)**	-	-	-	19.0	4.2	4.4	-	-
	**Other**	4.6	4.2	18.2	14.3	4.2	-	14.3	-
**Glume Color**	Green	100	95.8	100	83.3	100	100	90.5	100
	**Purple**	-	4.2	-	-	-	-	-	-
	**Purple (Green)**	-	-	-	4.2	-	-	9.5	-
	**Other**	-	-	-	12.50	-	-	-	-
**Leaf-Midrib Color**	**Purple (Green)**	-	-	-	12.5	-	-	20	-
	**White**	100	100	100	87.5	100	100	80	100
**Shoot Color**	**Purple (Green)**	-	4.2	-	16.7	4.2	-	9.5	-
	**Green**	100	95.8	100	83.3	95.8	100	90.5	100
**Cob Color**	Brown	5.4	-	-	8.9	8.1	2.8	-	-
	**Purple**	-	-	-	8.9	-	-	-	-
	**Red**	-	-	-	50	2.7	-	-	-
	**White**	94.6	100	100	32.2	9.2	97.2	100	100
**Presence of Dent**	**Yes**	22.2	-	11.1	11.1	27.8	88.9	22.2	16.7
	**No**	77.8	100	88.9	88.9	72.2	11.1	77.8	83.3

**Table 3:** Chi-square analysis of different qualitative traits evaluated across different environments of New Mexico.

	Los Lunas	Alcalde	Across Locations
**Tassel color**	131.12 ***	131.65 ***	260.23 ***
**Silk Color**	111.71 ***	122.89 ***	370.7 ***
**Glume Color**	160.47 ***	168.81 ***	476.5 ***
**Leaf Midrib Color**	85.17 ***	74.07 ***	159.0 ***
**Shoot Color**	81.38 ***	77.04 ***	158.3 ***
**Cob Color**	294.7 ***	229.18 ***	642.25 ***
**Presence of Dent**	47.68 ***	49.77 ***	99.55 ***

*** Significantly different at 0.001.

**Table 4:** Descriptive statistics of kernel biochemical traits evaluated across Los Lunas and Alcalde locations in New Mexico in 2014.

Landraces	Total Fatty Acids (%)	Protein (%)	Oil (%)	Starch (%)	Anthocyanin (mg/100 g)
**Navajo Blue**	5.1	10.9	5.3	63.3	65.3
**Santa Ana Blue**	6.3	11.4	6.5	63.4	70.2
**Los Lunas High**	6.2	10.3	6.4	63.5	61.7
**Flor Del Rio**	4.7	11.8	4.9	63.4	40.3
**Yoeme Blue**	6.3	11.6	6.5	59.6	61.5
**Ohio Blue**	5.4	11.2	5.6	65.8	63.8
**Hopi Blue**	6.1	11.0	6.3	63.3	67.2
**Taos Blue**	6.7	10.3	6.9	65.5	59.8
**Average**	5.8	11.0	6.1	63.4	61.2
**Range**	4.7–6.7	10.3–11.8	4.9–6.9	59.6–65.8	40.3–70.2
**LSD_0.05_**	2.50	1.73	2.59	4.90	20.98
**CV**	19.99	9.98	20.07	5.03	25.26

**Table 5:** Eigen value, variance contribution (%), and total cumulative variance (%) of principal components.

Principal Components	Eigen Value	Variance (%)	Cumulative Variance (%)
**1**	8.849	22.689	22.689
**2**	6.433	16.496	39.185
**3**	4.699	12.048	51.233
**4**	3.529	9.050	60.282
**5**	2.828	7.252	67.535
**6**	2.451	6.285	73.820
**7**	2.025	5.192	79.013
**8**	1.586	4.067	83.080
**9**	1.471	3.772	86.852
**10**	1.090	2.795	89.646
**11**	1.070	2.745	92.391
**12**	0.637	1.634	94.025
**13**	0.527	1.351	95.376
**14**	0.446	1.143	96.519
**15**	0.355	0.909	97.428
**16**	0.237	0.608	98.036
**17**	0.218	0.558	98.594
**18**	0.182	0.467	99.061
**19**	0.128	0.328	99.389
**20**	0.116	0.298	99.687
**21**	0.064	0.165	99.853
**22**	0.032	0.081	99.933
**23**	0.026	0.067	100.000

**Table 6:** Agro-morphological and biochemical trait contribution, correlation coefficient, and eigen vector for principle component 1 and 2.

Trait Category (Code)	Feature		R^2^		Eigenvector	
	PC1	PC2	PC1	PC2	1	2
**Plant Traits:**						
Ear Height	0.370	0.014	0.181	0.030	0.049	0.024
Plant Height	0.209	0.982	0.136	−0.251	0.027	−0.064
Ears/plant	1.926	2.803	0.413	−0.425	0.117	−0.085
Ear Placement Node	0.899	0.684	0.282	−0.210	0.079	−0.092
Leaves Above Primary Ear	1.784	0.009	0.397	0.025	0.087	0.025
Internodes	2.631	1.773	0.482	−0.338	0.150	−0.051
Nodes	2.631	1.773	0.482	−0.338	0.150	−0.051
Secondary Branches	0.068	0.214	0.077	−0.117	0.055	−0.065
Number of Tillers	5.476	0.030	−0.696	0.044	−0.216	0.020
**Ear Traits:**						
Circumference of Ear Top (ET)	1.125	6.217	0.316	−0.632	0.150	−0.215
Circumference of Ear Mid (EM)	8.770	0.225	0.881	0.120	0.292	0.050
Circumference of Ear Bottom (EB)	7.306	1.725	0.804	0.333	0.250	0.131
Ratio of ET/EB (ET_EB)	0.001	4.437	0.010	−0.534	0.005	−0.089
Ratio of EB/ET (EB_ET)	0.001	7.311	0.007	0.686	−0.045	0.252
Ear Length (EL)	0.554	4.890	−0.221	0.561	−0.087	0.301
Ear Diameter (ED)	8.776	0.318	0.881	0.143	0.293	0.086
Ear Weight (EW)	2.857	6.897	0.503	0.666	0.166	0.269
Circumference of Cob Top (CT)	5.251	0.528	0.682	−0.184	0.245	−0.125
Circumference of Cob Mid (CM)	6.378	0.006	0.751	−0.019	0.257	0.005
Circumference of Cob Bottom (CB)	3.158	0.044	0.529	0.053	0.191	0.005
Ratio of CT/CB (CT_CB)	3.134	0.964	0.527	−0.249	0.203	−0.159
Cob Diameter (CD)	1.639	1.496	0.381	−0.310	0.112	−0.080
Cob Weight (CW)	2.303	2.044	0.451	0.363	0.134	0.200
Ratio of ED/CD	2.744	2.351	0.493	0.389	0.153	0.160
Husk Leaves	1.487	0.176	−0.363	0.106	−0.186	0.053
**Kernel Traits:**						
Kernel Length (KL)	6.522	0.313	0.760	0.142	0.251	0.051
Kernel Width (KW)	5.223	1.110	0.680	−0.267	0.228	−0.101
Ratio of KL/KW (KL_KW)	0.032	2.259	0.053	−0.381	−0.011	−0.062
Ratio of KW/KL (KW_KL)	0.047	1.288	0.065	−0.288	0.026	−0.115
Kernels/Ear	0.008	6.914	0.026	0.667	−0.012	0.250
Kernel Weight/Ear	2.234	7.376	0.445	0.689	0.143	0.294
Kernel Weight	6.686	0.105	0.769	0.082	0.267	0.064
Kernel Rows	0.002	1.390	−0.012	0.299	0.010	0.159
100 Kernel Weight	5.662	0.925	0.708	0.244	0.246	0.097
**Biochemical Traits:**						
Total Fatty Acids	0.559	10.095	−0.222	0.806	−0.072	0.311
Protein	0.011	5.519	−0.032	−0.596	−0.011	−0.263
Oil	0.649	10.199	−0.240	0.810	−0.075	0.312
Starch	0.643	3.198	0.239	0.454	0.018	0.173
Anthocyanin	0.242	1.398	−0.146	0.300	−0.097	0.181

**Table 7:** Geographical and botanical information of eight different landraces of blue corn.

Accession	Altitude(m)	Source	Origin	Latitude	Longitude	Kernel	
						Color	Texture
**Navajo Blue**	1615	Plants of Southwest, Santa Fe, NM	Shiprock, NM	36	−112	Blue	Floury
**Santa Ana Blue**	1822	Agric.Sci.Center, Los Lunas. NM	Santa Ana Pueblo, NM	32	−108	Blue	Floury
**Los Lunas High**	1822	Agric.Sci.Center, Los Lunas. NM	Los Lunas, NM	32	−108	Blue	Floury
**Flor Del Rio**	1676	Native Seeds/SEARCH, Tuscon, AZ	Velarde, NM	36	−106	Red/Purple	Pop/Small Flint
**Yoeme Blue**	396	Native Seeds/SEARCH, Tuscon, AZ	Salt River Reservation, AZ	34	−112	Blue	Floury
**Ohio Blue**	310	Ohio State University, Wooster, OH	Wooster, OH	41	−82	Blue	Dent
**Hopi Blue**	1700	Native Seeds/SEARCH, Tuscon, AZ	Hopi Nation, AZ	35	−110	Blue	Floury
**Taos Blue**	2000	Native Seeds/SEARCH, Tuscon, AZ	Taos, NM	36	−106	Blue	Floury

## Data Availability

All the data is contained within the article and in the supplementary material.

## Supplementary Materials

The following are available online at https://www.mdpi.com/article/10.3390/ijms22073436/s1, Figure S1: Cross-validation error (CV error) for population structure K = 1 to K = 10, Figure S2: Population structure bar plots for K = 1 to K = 10, Figure S3: Growing region map of each blue maize landrace presented in this work, Table S1: Subsample contribution to each population structure for K = 1 to K = 10, Table S2: Descriptive statistics of plant traits evaluated at Los Lunas, New Mexico, Table S3: Descriptive statistics of plant traits evaluated at Alcalde, New Mexico, Table S4: Descriptive statistics of ear traits evaluated at Los Lunas, New Mexico, Table S5: Descriptive statistics of ear traits evaluated at Alcalde, New Mexico, Table S6: Descriptive statistics of kernel traits evaluated at Los Lunas, New Mexico, Table S7: Descriptive statistics of kernel traits evaluated at Alcalde, New Mexico, Table S8: Descriptive statistics of kernel biochemical traits evaluated at Los Lunas, New Mexico, Table S9: Descriptive statistics of kernel biochemical traits evaluated at Alcalde, New Mexico, Table S10: List of traits evaluated at pre and post-harvest stages.
