# Supplementary material for: Genotyping by Sequencing Reveals Genetic Relatedness of Southwestern U.S. Blue Maize Landraces
Source: Int J Mol Sci. 2021 Mar 26;22(7):3436. doi: 10.3390/ijms22073436 (PMC8037273; doi:10.3390/ijms22073436)
Supplement: Supplementary file 1 [file ijms-22-03436-s001.zip › Supplementary Info/Supplementary Table S2-S10. Descriptive stat of plant, ear, kernel and compositional traits (Nankar and Pratt, 03.18.2021).docx]

**Supplementary Information:**

**Table S2. Descriptive statistics of plant traits evaluated at Los Lunas, New Mexico**

|  | **Plant Height** | **Ear Height** | **Ears/ Plant** | **Number of Tillers** | **Secondary Branches** | **Leaves above Primary Ear** | **Number of Nodes** | **Number of Internodes** | **Ear Placement Node** |
| --- | --- | --- | --- | --- | --- | --- | --- | --- | --- |
| **Navajo Blue** | 144.3 | 60.6 | 2.1 | 2.2 | 12.3 | 5.8 | 12.3 | 11.3 | 5.7 |
| **Santa Clara Blue** | 161.1 | 73.0 | 2.3 | 2.9 | 13.0 | 5.8 | 13.2 | 12.2 | 6.2 |
| **Los Lunas High** | 133.6 | 62.3 | 2.0 | 0.9 | 13.3 | 5.6 | 11.8 | 10.8 | 5.3 |
| **Flor del Rio** | 148.7 | 69.8 | 2.3 | 3.5 | 15.6 | 6.3 | 12.4 | 11.4 | 5.6 |
| **Yoeme Blue** | 165.8 | 77.3 | 2.2 | 2.8 | 16.9 | 6.4 | 13.6 | 12.6 | 6.3 |
| **Ohio Blue** | 160.8 | 75.2 | 2.0 | 2.8 | 18.3 | 6.3 | 13.6 | 12.6 | 6.2 |
| **Hopi Blue** | 148.3 | 70.5 | 2.5 | 1.8 | 13.1 | 6.0 | 13.3 | 12.3 | 6.3 |
| **Taos Blue** | 151.3 | 74.9 | 1.0 | 3.1 | 17.8 | 5.8 | 12.5 | 11.5 | 5.8 |
| **Average** | 151.7 | 70.5 | 2.05 | 2.5 | 15.03 | 6.0 | 12.8 | 11.8 | 5.9 |
| **Range** | 133.6-165.8 | 60.6-77.3 | 1.0-2.5 | 0.9-3.5 | 12.3-18.3 | 5.6-6.4 | 11.8-13.6 | 10.8-12.6 | 5.3-6.3 |
| **LSD_0.05_** | 38.66 | 56.4 | 0.75 | 2.23 | 7.57 | 1.21 | 2.26 | 2.26 | 1.78 |

**Table S3. Descriptive statistics of plant traits evaluated at Alcalde, New Mexico**

|  | **Plant Height** | **Ear Height** | **Ears/ Plant** | **Number of Tillers** | **Secondary Branches** | **Leaves above Primary Ear** | **Number of Nodes** | **Number of Internodes** | **Ear Placement Node** |
| --- | --- | --- | --- | --- | --- | --- | --- | --- | --- |
| **Navajo Blue** | 207.3 | 87.3 | 1.9 | 2.9 | 22.3 | 6.8 | 15.1 | 14.1 | 7.3 |
| **Santa Clara Blue** | 240.2 | 106.3 | 1.9 | 4.7 | 16.8 | 6.8 | 14.7 | 13.7 | 7.0 |
| **Los Lunas High** | 222.1 | 101.0 | 2.0 | 3.9 | 19.9 | 7.1 | 15.8 | 14.8 | 7.8 |
| **Flor del Rio** | 196.3 | 96.6 | 1.7 | 3.9 | 18.6 | 6.4 | 14.5 | 13.5 | 7.1 |
| **Yoeme Blue** | 200.3 | 90.9 | 2.0 | 3.4 | 16.9 | 6.8 | 14.7 | 13.7 | 7.2 |
| **Ohio Blue** | 213.4 | 105.4 | 2.0 | 1.8 | 17.7 | 7.2 | 15.9 | 14.9 | 7.8 |
| **Hopi Blue** | 166.9 | 66.6 | 1.5 | 3.8 | 17.8 | 6.5 | 13.7 | 12.7 | 6.2 |
| **Taos Blue** | 224.6 | 94.0 | 1.8 | 4.0 | 23.0 | 6.7 | 15.1 | 14.1 | 7.1 |
| **Average** | 208.9 | 93.5 | 1.9 | 3.5 | 19.1 | 6.8 | 14.9 | 13.9 | 7.2 |
| **Range** | 166.9-240.2 | 66.6-106.3 | 1.5- 2.0 | 1.8- 4.7 | 16.8- 23.0 | 6.4- 7.2 | 13.7- 15.9 | 12.7- 14.9 | 6.2- 7.8 |
| **LSD_0.05_** | 56.42 | 24.75 | 0.57 | 1.46 | 3.45 | 0.74 | 1.60 | 1.60 | 1.27 |

**Table S4. Descriptive statistics of ear traits evaluated at Los Lunas, New Mexico**

| **Traits** | **Unit** | **Navajo Blue** | **Santa Clara Blue** | **Los Lunas High** | **Flor del Rio** | **Yoeme Blue** | **Ohio Blue** | **Hopi Blue** | **Taos Blue** | **Average** | **Range** | **LSD_0.05_** |
| --- | --- | --- | --- | --- | --- | --- | --- | --- | --- | --- | --- | --- |
| **ET** |  | 10.4 | 9.8 | 9.4 | 10.8 | 10.1 | 10.8 | 10.6 | 9.9 | 10.2 | 9.4-10.8 | 1.75 |
| **EM** |  | 13.1 | 12.5 | 12.6 | 12.3 | 12.6 | 13.8 | 12.6 | 12.4 | 12.7 | 12.3-13.8 | 0.74 |
| **EB** |  | 13.9 | 13.6 | 13.4 | 13.3 | 13.2 | 14.8 | 13.1 | 13.5 | 13.6 | 13.1-14.8 | 0.81 |
| **CT** |  | 6.5 | 6.2 | 6.6 | 6.4 | 6.7 | 6.4 | 6.2 | 6.0 | 6.4 | 6.2-6.7 | 0.68 |
| **CM** | cm | 8.2 | 8.1 | 8.4 | 8.0 | 8.3 | 8.5 | 8.4 | 8.0 | 8.2 | 8.0-8.5 | 0.59 |
| **CB** |  | 9.2 | 9.4 | 9.1 | 8.9 | 9.0 | 9.5 | 9.2 | 9.0 | 9.1 | 8.9-9.4 | 0.53 |
| **EL** |  | 22.8 | 23.4 | 22.1 | 20.4 | 20.9 | 22.0 | 21.9 | 21.5 | 21.9 | 20.4-22.8 | 2.31 |
| **ED** |  | 3.9 | 5.4 | 3.9 | 3.7 | 3.7 | 4.1 | 3.9 | 3.8 | 4.0 | 3.7-5.4 | 1.77 |
| **CD** |  | 2.7 | 2.6 | 2.8 | 2.6 | 2.7 | 2.8 | 2.8 | 2.6 | 2.7 | 2.6-2.8 | 0.15 |
| **ET/EB** |  | 0.73 | 0.71 | 0.68 | 0.78 | 0.75 | 0.72 | 0.81 | 0.78 | 0.74 | 0.68-0.81 | 0.09 |
| **CT/CB** |  | 0.71 | 0.66 | 0.72 | 0.72 | 0.74 | 0.67 | 0.68 | 0.66 | 0.70 | 0.66-0.74 | 0.07 |
| **ED/CD** |  | 1.47 | 2.04 | 1.40 | 1.40 | 1.37 | 1.46 | 1.40 | 1.44 | 1.50 | 1.37-2.04 | 0.66 |
| **EW** | g | 177.1 | 169.4 | 148.4 | 142.7 | 136.0 | 191.3 | 148.8 | 156.5 | 158.8 | 136.0-191.3 | 30.58 |
| **CW** | g | 33.2 | 32.8 | 27.1 | 27.4 | 23.3 | 32.7 | 29.7 | 25.3 | 29.0 | 23.3-33.2 | 6.25 |
| **Number of Husks** |  | 11.2 | 11.1 | 10.9 | 10.1 | 11.2 | 12.1 | 11.8 | 11.3 | 11.2 | 10.1-12.1 | 2.70 |
| **Kernel Rows/Ear** |  | 13.7 | 14.6 | 13.7 | 14.4 | 14.1 | 15.2 | 12.3 | 14.2 | 14.0 | 12.3-15.2 | 2.21 |
| **Number of Kernels/Row** |  | 41.9 | 43.6 | 38.7 | 38.8 | 31.2 | 39.8 | 38.7 | 38.6 | 38.9 | 31.2-41.9 | 4.63 |
| **Number of kernels/Ear** |  | 501.8 | 573.1 | 454.0 | 494.1 | 380.9 | 522.2 | 439.7 | 469.7 | 479.4 | 380.9-573.1 | 78.13 |

**Table S5. Descriptive statistics of ear traits evaluated at Alcalde, New Mexico**

| **Traits** | **Unit** | **Navajo Blue** | **Santa Clara Blue** | **Los Lunas High** | **Flor del Rio** | **Yoeme Blue** | **Ohio Blue** | **Hopi Blue** | **Taos Blue** | **Average** | **Range** | **LSD_0.05_** |
| --- | --- | --- | --- | --- | --- | --- | --- | --- | --- | --- | --- | --- |
| **ET** |  | 10.0 | 8.3 | 9.6 | 10.0 | 7.0 | 10.4 | 9.8 | 8.0 | 9.1 | 8.0-10.4 | 2.20 |
| **EM** |  | 13.0 | 12.3 | 13.4 | 13.0 | 13.0 | 14.3 | 12.9 | 13.0 | 13.1 | 12.3-14.3 | 0.97 |
| **EB** |  | 13.5 | 12.9 | 13.9 | 13.5 | 13.6 | 14.7 | 13.5 | 13.6 | 13.6 | 12.9-14.7 | 1.12 |
| **CT** |  | 5.8 | 5.2 | 6.5 | 5.9 | 5.7 | 6.6 | 5.9 | 5.8 | 5.9 | 5.2-6.6 | 1.11 |
| **CM** | cm | 8.6 | 7.9 | 8.5 | 8.1 | 8.3 | 8.8 | 8.4 | 7.9 | 8.3 | 7.9-8.8 | 0.44 |
| **CB** |  | 9.6 | 9.1 | 9.6 | 9.1 | 9.4 | 9.7 | 9.7 | 8.9 | 9.4 | 9.1-9.7 | 0.38 |
| **EL** |  | 22.3 | 24.1 | 21.3 | 20.4 | 22.9 | 20.6 | 22.1 | 22.6 | 22.0 | 20.4-22.9 | 3.87 |
| **ED** |  | 4.0 | 3.7 | 4.0 | 3.9 | 3.9 | 4.4 | 3.8 | 4.0 | 4.0 | 3.7-4.4 | 0.26 |
| **CD** |  | 2.7 | 2.6 | 2.7 | 2.6 | 2.7 | 2.7 | 2.7 | 2.5 | 2.6 | 2.5-2.7 | 0.19 |
| **ET/EB** |  | 0.74 | 0.64 | 0.69 | 0.74 | 0.51 | 0.70 | 0.73 | 0.59 | 0.67 | 0.51-0.74 | 0.20 |
| **CT/CB** |  | 0.60 | 0.57 | 0.68 | 0.65 | 0.61 | 0.68 | 0.61 | 0.65 | 0.63 | 0.57-0.68 | 0.12 |
| **ED/CD** |  | 1.49 | 1.45 | 1.50 | 1.51 | 1.47 | 1.65 | 1.43 | 1.58 | 1.51 | 1.42-1.65 | 0.13 |
| **EW** | g | 174.6 | 155.3 | 165.9 | 160.9 | 161.9 | 186.5 | 159.0 | 176.7 | 167.6 | 155.3-186.5 | 48.68 |
| **CW** | g | 34.3 | 32.3 | 34.9 | 29.6 | 32.8 | 35.0 | 32.6 | 27.6 | 32.4 | 27.6-35.0 | 9.28 |
| **Number of Husks** |  | 9.3 | 11.2 | 9.5 | 7.3 | 9.6 | 10.0 | 9.7 | 10.8 | 9.7 | 7.3-11.2 | 2.68 |
| **Kernel Rows/Ear** |  | 12.7 | 14.4 | 14.7 | 14.0 | 13.8 | 15.3 | 15.0 | 15.0 | 14.3 | 12.7-15.3 | 1.05 |
| **Number of Kernels/Row** |  | 40.3 | 41.9 | 38.6 | 38.3 | 41.4 | 38.7 | 37.7 | 40.7 | 39.7 | 37.7-41.9 | 7.31 |
| **Number of kernels/Ear** |  | 447.8 | 528.5 | 524.1 | 496.7 | 493.2 | 517.2 | 479.8 | 559.9 | 505.8 | 447.8-559.9 | 104.7 |

**Where,**

**ET =** Circumference of Ear Top

**EM =** Circumference of Ear Mid

**EB =** Circumference of Ear Bottom

**ET/EB =** Circumference of Ear Top/Circumference Ear Bottom

**CT =** Circumference of Cob Top

**CM =** Circumference of Cob Mid

**CB =** Circumference of Cob Bottom

**CT/CB =** Circumference of Cob Top/Circumference of Cob Bottom

**Table S6. Descriptive statistics of kernel traits evaluated at Los Lunas, New Mexico**

|  | **KL** | **KW** | **KL/KW** | **KW/KL** | **Kernel Weight** | **100-Kernel Weight** | **Grain Yield** |
| --- | --- | --- | --- | --- | --- | --- | --- |
|  | **(cm)** | |  |  | **(g)** | | **(mg/ha)** |
| **Navajo Blue** | 1.16 | 0.86 | 1.37 | 0.75 | 0.30 | 29.3 | 3.19 |
| **Santa Clara Blue** | 1.07 | 0.75 | 1.44 | 0.70 | 0.24 | 23.0 | 2.81 |
| **Los Lunas High** | 1.08 | 0.82 | 1.33 | 0.76 | 0.27 | 26.1 | 2.34 |
| **Los Lunas** | 1.09 | 0.77 | 1.43 | 0.71 | 0.23 | 23.3 | 2.75 |
| **Yoeme Blue** | 1.07 | 0.77 | 1.42 | 0.72 | 0.23 | 23.5 | 1.56 |
| **Ohio Blue** | 1.23 | 0.85 | 1.47 | 0.69 | 0.30 | 30.1 | 3.30 |
| **Hopi Blue** | 1.09 | 0.89 | 1.24 | 0.82 | 0.30 | 29.3 | 2.53 |
| **Taos Blue** | 1.12 | 0.78 | 1.44 | 0.70 | 0.26 | 25.8 | 2.76 |
| **Average** | 1.11 | 0.81 | 1.39 | 0.73 | 0.27 | 26.3 | 2.65 |
| **Range** | 1.07-1.23 | 0.77-0.89 | 1.24-1.47 | 0.69-0.82 | 0.23-0.30 | 23.0-30.1 | 1.56-3.19 |
| **LSD_0.05_** | 0.097 | 0.11 | 0.14 | 0.05 | 0.05 | 4.30 | 1.16 |

**Table S7. Descriptive statistics of kernel traits evaluated at Alcalde, New Mexico**

|  | **KL** | **KW** | **KL/KW** | **KW/KL** | **Kernel Weight** | **100-Kernel Weight** | **Grain Yield** |
| --- | --- | --- | --- | --- | --- | --- | --- |
|  | **(cm)** | |  |  | **(g)** | | **(mg/ha)** |
| **Navajo Blue** | 1.13 | 0.86 | 1.24 | 0.76 | 0.31 | 30.4 | 3.25 |
| **Santa Clara Blue** | 1.04 | 0.74 | 1.30 | 0.71 | 0.24 | 24.2 | 2.82 |
| **Los Lunas High** | 1.12 | 0.79 | 1.28 | 0.71 | 0.26 | 25.4 | 3.31 |
| **Los Lunas** | 1.11 | 0.77 | 1.33 | 0.70 | 0.28 | 26.5 | 3.11 |
| **Yoeme Blue** | 1.08 | 0.79 | 1.26 | 0.73 | 0.27 | 27.4 | 4.10 |
| **Ohio Blue** | 1.21 | 0.82 | 1.39 | 0.68 | 0.30 | 29.6 | 3.83 |
| **Hopi Blue** | 1.06 | 0.78 | 1.26 | 0.73 | 0.27 | 26.8 | 2.97 |
| **Taos Blue** | 1.13 | 0.75 | 1.38 | 0.66 | 0.28 | 26.7 | 3.60 |
| **Average** | 1.11 | 0.79 | 1.31 | 0.71 | 0.28 | 27.1 | 3.37 |
| **Range** | 1.04-1.21 | 0.74-0.86 | 1.24-1.39 | 0.66-0.76 | 0.24-0.31 | 24.2-30.4 | 2.82-3.60 |
| **LSD_0.05_** | 0.11 | 0.05 | 0.14 | 0.06 | 0.03 | 3.39 | 0.98 |

**Table S8. Descriptive statistics of kernel biochemical traits evaluated at Los Lunas, New Mexico**

| **Accessions** | **Total Fatty Acids** | **Protein** | **Oil** | **Starch** | **Anthocyanin** |
| --- | --- | --- | --- | --- | --- |
|  | **(%)** | | | | **(mg/100g)** |
| **Navajo Blue** | 5.10 | 12.1 | 5.29 | 62.5 | 55.1 |
| **Santa Clara Blue** | 6.36 | 12.0 | 6.59 | 63.0 | 94.7 |
| **Los Lunas High** | 6.23 | 11.5 | 6.47 | 62.1 | 62.6 |
| **Flor Del Rio** | 4.82 | 13.1 | 5.01 | 62.5 | 63.2 |
| **Yoeme Blue** | 6.15 | 11.9 | 6.37 | 59.8 | 75.1 |
| **Ohio Blue** | 5.42 | 11.9 | 5.62 | 65.4 | 78.2 |
| **Hopi Blue** | 6.20 | 11.5 | 6.42 | 61.1 | 79.9 |
| **Taos Blue** | 6.54 | 10.3 | 6.78 | 67.1 | 79.1 |
| **Average** | 5.85 | 11.78 | 6.07 | 62.9 | 73.5 |
| **Range** | 4.82-6.54 | 10.3-13.1 | 5.01-6.78 | 61.1-67.1 | 55.1-94.7 |
| **LSD_0.05_** | 1.17 | 1.98 | 1.22 | 4.69 | 32.02 |

**Table S9. Descriptive statistics of kernel biochemical traits evaluated at Alcalde, New Mexico**

| **Accessions** | **Total Fatty Acids** | **Protein** | **Oil** | **Starch** | **Anthocyanin** |
| --- | --- | --- | --- | --- | --- |
|  | **(%)** | | | | **(mg/100g)** |
| **Navajo Blue** | 5.04 | 9.62 | 5.22 | 64.0 | 75.5 |
| **Santa Clara Blue** | 6.20 | 10.7 | 6.42 | 63.8 | 45.6 |
| **Los Lunas High** | 6.18 | 9.14 | 6.40 | 64.9 | 60.8 |
| **Flor Del Rio** | 4.59 | 10.4 | 4.76 | 64.2 | 17.4 |
| **Yoeme Blue** | 6.47 | 11.3 | 6.72 | 59.3 | 47.9 |
| **Ohio Blue** | 5.31 | 10.5 | 5.51 | 66.1 | 49.4 |
| **Hopi Blue** | 6.04 | 10.4 | 6.27 | 65.4 | 54.5 |
| **Taos Blue** | 6.81 | 10.3 | 7.07 | 63.8 | 40.5 |
| **Average** | 5.83 | 10.29 | 6.05 | 63.9 | 48.9 |
| **Range** | 4.59-6.81 | 9.62-11.3 | 4.76-7.07 | 59.3-66.1 | 17.4-75.5 |
| **LSD_0.05_** | 2.50 | 1.73 | 2.59 | 4.90 | 20.98 |

**Table S10. List of traits evaluated at pre and post-harvest stages**

| **Trait** | **Unit** |
| --- | --- |
| **Pre-Harvest** |  |
| **Plant Traits:** |  |
| Plant height | cm |
| Ear height | cm |
| Number of ears/plant |  |
| Number of tillers/plant |  |
| Number of secondary branches/plant |  |
| Color of shoot |  |
| Color of midrib |  |
| Color of tassel |  |
| Color of silk |  |
| Color of glume |  |
| Number of leaves above primary ear |  |
| Number of nodes |  |
| Number of internodes |  |
| Number of node at primary ear |  |
| **Post-Harvest** |  |
| **Ear Traits:** |  |
| Circumference of ear top, mid and bottom | cm |
| Ratio of ear top and ear bottom |  |
| Circumference of cob top, mid and bottom | cm |
| Ratio of cob top and bottom |  |
| Number of husk leaves |  |
| Ear length | cm |
| Ear diameter | cm |
| Ear weight | g |
| Cob diameter | cm |
| Cob weight | g |
| Ratio of ear diameter and cob diameter |  |
| Presence of dent |  |
| Number of kernel rows/Ear |  |
| Number of kernels/Row  Number of kernels/Ear |  |
| **Kernel Traits:** |  |
| Kernel length | cm |
| Kernel width | cm |
| Ratio of kernel length and kernel width |  |
| Ratio of kernel width and kernel length |  |
| Kernel weight | g |
| 100 kernel weight | g |
| Grain yield | mg/ha |
| **Kernel Biochemical Traits:** |  |
| Total Fatty Acids | % |
| Protein | % |
| Oil | % |
| Starch | % |
| Anthocyanin | mg/100g |
