# Supplementary figures and images for: Genotyping by Sequencing Reveals Genetic Relatedness of Southwestern U.S. Blue Maize Landraces
Source: Int J Mol Sci. 2021 Mar 26;22(7):3436. doi: 10.3390/ijms22073436 (PMC8037273; doi:10.3390/ijms22073436)

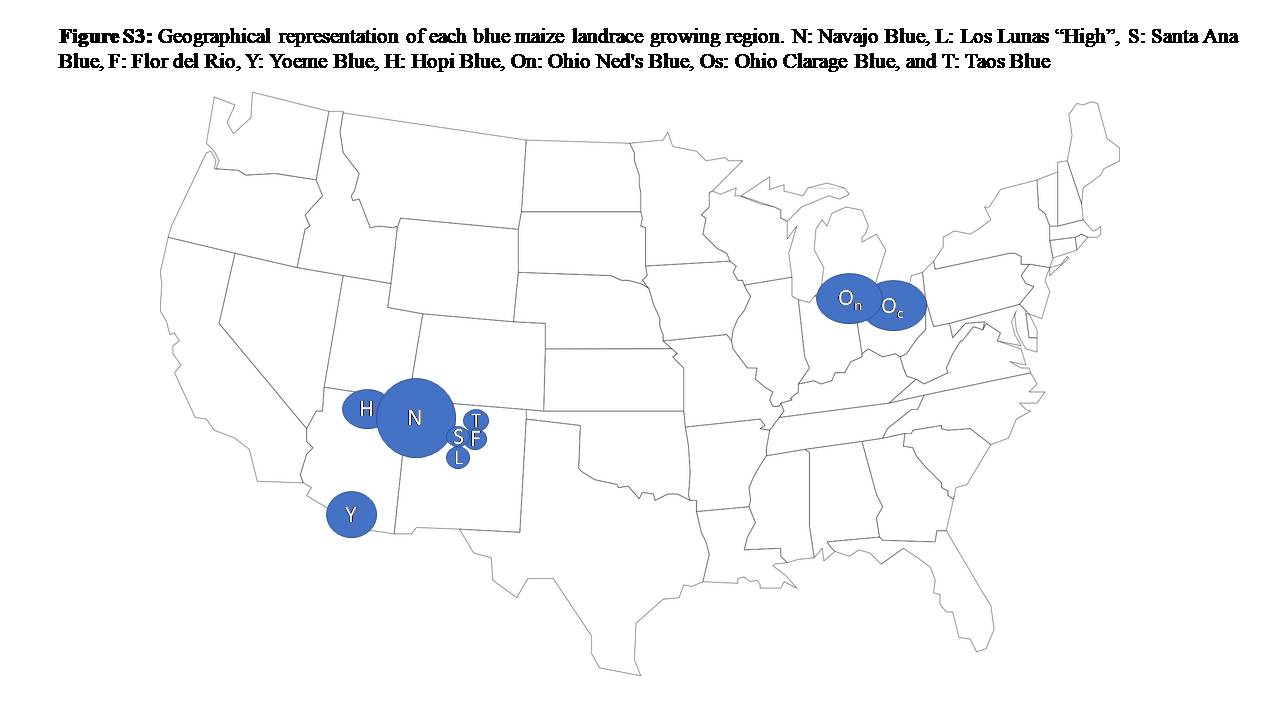

Supplement: Supplementary file 1 [file ijms-22-03436-s001.zip › Supplementary Info/Supplementary Figure 3. Growing region map of studied blue maize landraces (Nankar and Pratt, 03.18.2021).JPG]
